# Closure of Varicella-Zoster Virus–Containing Vaccines Pregnancy Registry — United States, 2013

**Published:** 2014-08-22

**Authors:** Mona Marin, English D. Willis, Ann Marko, Sonja A. Rasmussen, Stephanie R. Bialek, Adrian Dana

**Affiliations:** 1 Division of Viral Diseases, National Center for Immunization and Respiratory Diseases, CDC; 2 Clinical Safety and Risk Management, Merck & Co., Inc.; 3 Influenza Coordination Unit, Office of Infectious Diseases, CDC

Vaccines that contain live attenuated varicella-zoster virus (VZV) (Varivax, ProQuad, and Zostavax [all products of Merck & Co., Inc.]) are contraindicated during pregnancy (1,2). To monitor the pregnancy outcomes of women inadvertently vaccinated with VZV-containing vaccines immediately before or during pregnancy, Merck and CDC established the Merck/CDC Pregnancy Registry for VZV-Containing Vaccines in 1995 (3). This report updates previously published summaries of registry data (4,5), provides the rationale for the closure of the registry, and describes plans for continued monitoring of the safety of these vaccines when inadvertently administered to pregnant women or immediately before pregnancy. From inception of the registry in 1995 through March 2012, no cases of congenital varicella syndrome and no increased prevalence of other birth defects have been detected among women vaccinated within 3 months before or during pregnancy. Although a small risk for congenital varicella syndrome cannot be ruled out, the number of exposures being registered each year (approximately two varicella-susceptible women exposed during the high-risk period for congenital varicella syndrome) is now too low to improve on the current estimate of the risk.

Congenital varicella syndrome is characterized by cutaneous scarring and/or limb hypoplasia; other associated anomalies include microcephaly, muscular atrophy, ocular or neurologic abnormalities, and low birth weight. Because exposure to wild-type VZV in utero might result in congenital varicella syndrome, vaccines that contain live, attenuated VZV are contraindicated during pregnancy. To monitor the pregnancy outcomes of women inadvertently vaccinated with VZV-containing vaccines immediately before or during pregnancy, Merck, in collaboration with CDC, established a registry in 1995, when Varivax, indicated for prevention of varicella (chickenpox) in persons aged ≥12 months, was licensed in the United States (1,3). Reports of exposure to ProQuad, which is indicated for simultaneous vaccination against measles, mumps, rubella, and varicella among children aged 12 months through 12 years, and Zostavax, which is licensed for the prevention of herpes zoster (shingles) among persons aged ≥50 years, were added to the registry in 2006, upon licensure of those vaccines. Detailed methods for the pregnancy registry have been described previously (4,5).

From March 1995 to March 2012, the registry received 860 prospective reports (received before the outcome of pregnancy was known) and 68 retrospective reports (received after the outcome of pregnancy was known) of women who inadvertently received Varivax within 3 months before pregnancy or at any time during pregnancy, and whose pregnancy outcomes were known, available for analysis, and considered complete. No defects consistent with congenital varicella syndrome were reported among the live-born infants or any of the conceptuses lost because of spontaneous abortion or elective termination for which information was available. Based on the 95 live-born infants of varicella-susceptible women exposed during the high-risk period for congenital varicella syndrome (first and second trimester of pregnancy) who were reported prospectively to the registry, the 95% confidence interval for risk for congenital varicella syndrome ranged from 0% to 3.8%. The overall prevalence for major birth defects in the registry was 2.2% among live-born infants (95% confidence interval = 1.3–3.5), similar to the prevalence in the general population (6). These data are reassuring regarding the safety of Varivax inadvertently administered during pregnancy; however, the number of women enrolled is insufficient to exclude a theoretical risk for congenital varicella syndrome lower than the risk estimated after infection with wild-type VZV (approximately 1% of live births when infection is contracted during the first two trimesters of pregnancy) (7). No informative data on outcomes after exposures to ProQuad or Zostavax during pregnancy were obtained. Neither vaccine is licensed for the age groups that include women of traditional childbearing ages. Only nine reports of exposure to these vaccines were received by the registry since 2006. The annual reports with detailed data are available to health care providers from the manufacturer upon request (telephone, 1-800-986-8999).

As a result of sustained high coverage with varicella vaccine in childhood, and because VZV-containing vaccines are contraindicated during pregnancy, the number of vaccine administrations (inadvertent) immediately before and during pregnancy, and thus registry enrollments, have declined. The number of varicella-susceptible women exposed during the high risk-period for congenital varicella syndrome decreased to a yearly average of two during 2009–2012. To lower the estimate of the theoretical risk for congenital varicella syndrome among varicella-susceptible women exposed to Varivax during the high-risk period from the current 95% confidence interval upper bound estimate of 3.8% to 1.0% (the risk after infection with wild-type VZV), an additional 271 exposed susceptible women would need to be enrolled. At the observed average rate of annual enrollment, that number would not be reached until the year 2147.

The low rate of exposure of varicella-susceptible women of childbearing age to VZV-containing vaccines, in addition to the rarity of the outcome, contribute to the low feasibility that the registry will provide more robust data on the risk for congenital varicella syndrome within a reasonable timeframe. For this reason, the Food and Drug Administration, in support of the closure of the registry, approved the revision of information in the product labels regarding the registry (8). New patient enrollment was discontinued as of October 16, 2013. Follow-up of patients enrolled before this date will continue until after their estimated date of delivery (after July 2014), and final data will be analyzed for a summary report.

Because a theoretical risk for congenital varicella syndrome cannot be ruled out, pregnant women should not be vaccinated with Varivax, ProQuad, or Zostavax. The Advisory Committee on Immunization Practices also recommends that women should be counseled to avoid becoming pregnant for 1 month after each dose of a VZV-containing vaccine, considering the biologic plausibility of vaccine virus replication (1,2).

Merck will continue to monitor pregnancy outcomes after inadvertent exposures to VZV-containing vaccines during pregnancy or within 3 months before conception. CDC and the Food and Drug Administration will continue to monitor adverse events after vaccination with VZV-containing vaccines through the Vaccine Adverse Event Reporting System (VAERS). New cases of exposure immediately before or during pregnancy or other adverse events after vaccination with Varivax, ProQuad, or Zostavax, should be reported to Merck (telephone, 1-877-888-4231) and to VAERS (https://vaers.hhs.gov/index ). Laboratory testing and strain identification for VZV for any suspected pregnancy-related vaccine adverse event will continue to be provided, if requested, by CDC (additional information available at http://www.cdc.gov/chickenpox/lab-testing/collecting-specimens.html ) and through Merck’s VZV-identification program (telephone, 1-877-888-4231).
